# Job Strain and Trajectories of Cognitive Change Before and After Retirement

**DOI:** 10.1093/geronb/gbab033

**Published:** 2021-02-24

**Authors:** Charlotta Nilsen, Monica E Nelson, Ross Andel, Michael Crowe, Deborah Finkel, Nancy L Pedersen

**Affiliations:** 1 Aging Research Center, Karolinska Institutet/Stockholm University, Stockholm, Sweden; 2 Stress Research Institute, Stockholm University, Stockholm, Sweden; 3 School of Aging Studies, University of South Florida, Tampa, USA; 4 Department of Neurology, 2nd Faculty of Medicine, Charles University and Motol University Hospital, Prague, Czech Republic; 5 Department of Psychology, University of Alabama at Birmingham, USA; 6 Institute for Gerontology, Jönköping University, Jönköping, Sweden; 7 Department of Psychology, Indiana University Southeast, New Albany, USA; 8 Department of Medical Epidemiology and Biostatistics, Karolinska Institutet, Stockholm, Sweden; 9 Department of Psychology, University of Southern California, Los Angeles, USA

**Keywords:** Cognitive aging, Multiple cognitive domains, Postretirement change, Preretirement change, Work-related stress

## Abstract

**Objectives:**

We examined associations between job strain and trajectories of change in cognitive functioning (general cognitive ability plus verbal, spatial, memory, and speed domains) before and after retirement.

**Methods:**

Data on indicators of job strain, retirement age, and cognitive factors were available from 307 members of the Swedish Adoption/Twin Study of Aging. Participants were followed up for up to 27 years (mean = 15.4, *SD* = 8.5).

**Results:**

In growth curve analyses controlling for age, sex, education, depressive symptoms, cardiovascular health, and twinness, greater job strain was associated with general cognitive ability (estimate = −1.33, p = .002), worse memory (estimate = −1.22, *p* = .007), speed (estimate = −1.11, *p* = .012), and spatial ability (estimate = −0.96, *p* = .043) at retirement. Greater job strain was also associated with less improvement in general cognitive ability before retirement and a somewhat slower decline after retirement. The sex-stratified analyses showed that the smaller gains of general cognitive ability before retirement (estimate = −1.09, *p* = .005) were only observed in women. Domain-specific analyses revealed that greater job strain was associated with less improvement in spatial (estimate = −1.35, *p* = .010) and verbal (estimate = −0.64, *p* = .047) ability before retirement in women and a slower decline in memory after retirement in women (estimate = 0.85, *p* = .008) and men (estimate = 1.12, *p* = .013). Neither preretirement nor postretirement speed was affected significantly by job strain.

**Discussion:**

Greater job strain may have a negative influence on overall cognitive functioning prior to and at retirement, while interrupting exposure to job strain (postretirement) may slow the rate of cognitive aging. Reducing the level of stress at work should be seen as a potential target for intervention to improve cognitive aging outcomes.

Work environment plays an important role in health and aging, with research consistently attributing late-life aging outcomes at least partially to differences in the work environment. This is not surprising given the amount of time people generally spend working over the course of their lives. Work-related stress is one aspect of the work environment that has been receiving increased attention (Andel et al., 2015; Fratiglioni et al., 2020; Nilsen et al., 2019; Pan et al., 2019; Sabbath et al., 2016; Sindi et al., 2016), with growing evidence suggesting that work-related stress may have an influence on the aging process.

Work-related stress is one type of chronic stress, which is thought to accelerate the aging process (Finch & Seeman, 1999), likely due to its adverse effects on neuronal structures (Radley et al., 2004), particularly in the hippocampal region (Sapolsky, 1996). For example, work objectively characterized as stressful was found to activate the endocrine system in a manner typical of the fight-or-flight response (Häusser et al., 2011).

The job strain model of stress (Karasek, 1979) is one established framework for assessing work-related stress. It posits that low control, high demands, and the negative combination of these indicators (i.e., job strain—low job control combined with high job demands) have adverse effects on health, such as an increased risk of cardiovascular disease (CVD; Nyberg et al., 2013), diabetes (Mutambudzi & Javed, 2016), insomnia (Yang et al., 2018), and depressive symptoms (Mezuk et al., 2011).

The job strain model has also been applied to the study of cognitive health. Low job control and greater job strain have been associated with both reduced cognitive function (Andel et al., 2015; Nexø et al., 2016; Pan et al., 2019; Sabbath et al., 2016; Then et al., 2014) and dementia (Fratiglioni et al., 2020; Sindi et al., 2016; Then et al., 2014). Inconclusive results tend to be reported for job demands (Nexø et al., 2016; Nilsen et al., 2014; Then et al., 2014), possibly due to the interactive nature of job demands with mentally stimulating work.

Stress, including work-related stress, may be different for women and men, potentially leading to gender differences. For example, experiencing stressful life events in midlife was recently related to a greater memory decline in women than men (Munro et al., 2019). Therefore, despite some research suggesting a lack of differences between women and men in cognitive aging trajectories (Finkel et al., 2006), sex-specific exposure to work-related stress may still reflect differences in cognitive aging.

While there is some evidence for the association between work-related stress and subsequent level of cognition and risk of dementia, less is known about how specifically work-related stress may affect the trajectory of cognitive aging. Few studies investigated job strain in relation to cognitive change. Pan et al. (2019) found job strain to be associated with a faster global cognitive decline. Elovainio et al. (2009) found no significant association between job strain and change in vocabulary and phonemic fluency, while Agbenyikey et al. (2015) found job strain to be associated with a decline in word recognition skills, verbal learning, and memory.

A related area of research involves examining the association between retirement and subsequent cognitive performance. This research builds on the idea that work, regardless of job characteristics, offers sense of purpose, daily structure, social interaction, and mental activity that may help support cognitive function with age. Removal of these positive aspects of work at retirement may lead to worsened cognitive functioning above and beyond the effect of age alone (Bonsang et al., 2012; Coe et al., 2012; Rohwedder & Willis, 2010). However, some results do not support this notion (Meng et al., 2017). One possibility to build on retirement research is to consider work characteristics.

However, little is known about how the association between job strain and cognitive change may be influenced by retirement. So far, to the best of our knowledge, only Andel et al. (2015) investigated the association between job strain and change in episodic memory before and after retirement. Reported age at retirement was used as a pivot point, thus better tapping into the impact of direct exposure to job strain. They found that high job strain was not significantly associated with episodic memory before retirement, but it was associated with an accelerated rate of decline after retirement. One potential explanation for the finding is the fact that cognitive aging in some domains typically does not become apparent until after traditional retirement ages (Schaie, 2005). However, the study only included a phone-based assessment of episodic memory rather than a comprehensive set of cognitive tests.

We build on previous research by using data from the Swedish Adoption/Twin Study of Aging (SATSA) to examine job strain and cognitive change before and after retirement, using nine in-person testing (IPT) sessions across 27 years of follow-up. Ours is the first study to explore this question with a comprehensive assessment of cognitive function with multiple waves of in-person data collection across four domains (verbal, spatial, memory, and speed) and general cognitive ability. Based on existing evidence, we hypothesized that individuals with greater job strain would show more negative cognitive aging outcomes, including poorer cognitive function at retirement and an accelerated age-related cognitive decline. Given that both cognitive outcomes and work characteristics often differ by gender (Munro et al., 2019; Theorell et al., 2014), we also considered potential differences in the association between job strain and cognitive aging in sex-stratified analyses.

## Method

### Participants

Ascertainment procedures for SATSA have been described previously (Finkel & Pedersen, 2004). In brief, the sample is a subset of twins from the population-based Swedish Twin Registry. The base population comprises all pairs of twins who indicated that they had been separated before the age of 11 and reared apart, and a sample of twins reared together matched on the basis of gender and date and county of birth. Twins were mailed questionnaires in 1984 and a sample of those pairs aged 50 years or older in which both twins responded was invited to participate in an additional in-person examination of health and cognitive abilities. IPT was performed nine times between 1986 and 2014 and took place in a location convenient to the twins. Testing was completed during a single 4-h visit. Intervals between testing sessions ranged from 2 to 7 years; the total time span from IPT1 to IPT10 was 27 years (note that IPT4 had a reduced sample due to limited funding for in-person visits). In all, 851 individuals had cognitive data available from at least one testing occasion, of whom 491 also had valid information about their wage-earning occupation. Note that those working on their own farm, those reporting being a housewife, and similar nonwage-earning occupations do not have job strain codes. These 360 without gainful occupation were about 3 years older at baseline than the 491 with a valid occupation (65.5 ± 8.6 years vs. 62.1 ± 8.5 years) and were more likely to be women (65% women vs. 55% women). Among the 491 individuals, 126 entered the study more than 4 years (more than one wave) after retirement, leaving a sample of 365. Dementia status, determined by clinical diagnosis based on well-established diagnostic criteria (Gatz et al., 1997), was used as an exclusion criterion for the current analyses. Of the 365, 58 developed dementia, resulting in an analytic sample of 307 nondemented participants. Among the 544 excluded participants, 360 were not gainfully employed, 126 retired too early to be included in analyses, and 58 developed dementia. Those excluded were older (66.6 ± 8.5 vs. 58.2 ± 6.1, *t*[849] = 15.2, *p* < .001) and more likely to be women (64% vs. 52%, χ ^2^(1, *n* = 851) = 11.7, *p* < .001).

The number of participants in each wave is given in Table 1 along with the mean age at each wave. The number of participants and mean age at each wave did not change monotonically because SATSA continued to add participants at any wave at which cognitive data were collected until IPT5. Participants contributed up to 1,710 observations to the analyses of each cognitive measure. Overall, 262 of the 307 participants (85%) were tested at least three times over the course of the study, with 54 (18%) tested for cognitive performance in all nine waves.

**Table 1.** Number of Participants in Each Wave

Wave	Memory	Speed	Verbal	Spatial	Overall	Age^a^
IPT1	177	175	177	174	173	61.1 (4.9)
IPT2	198	194	190	189	177	61.0 (6.3)
IPT3	203	196	202	202	194	63.0 (7.0)
IPT4	20	21	21	20	19	64.8 (7.4)
IPT5	231	233	234	226	224	66.6 (8.0)
IPT6	210	214	214	199	199	69.2 (7.6)
IPT7	179	192	193	170	169	71.9 (7.6)
IPT8	156	175	175	134	132	74.0 (7.5)
IPT9	154	158	158	142	138	75.5 (7.3)
IPT10	140	144	146	121	119	77.1 (7.0)
Total number of observations	1,668	1,702	1,710	1,577	1,544	—

*Note:* IPT = in-person testing.

^a^Age is represented as mean (*SD*).

Post hoc power calculations were conducted in G*Power separately for women and men with job strain defined as a dichotomous variable. For women (*n* = 159), the power for the assessment of the cross-sectional association between job strain and cognition revealed a power estimate of 0.99 and for the longitudinal association between job strain and cognition revealed a power estimate of 1.00. For men (*n* = 148), the power for the assessment of the cross-sectional association between job strain and cognition revealed a power estimate of 0.98 and for the longitudinal association between job strain and cognition revealed a power estimate of 1.00.

### Measures

#### Dependent variables: cognitive components

Four cognitive domains are represented in the SATSA cognitive test battery (Pedersen et al., 1992) to test verbal ability, spatial ability, memory, speed, and general cognitive ability. Verbal abilities are tapped by tests of information from the Wechsler Adult Intelligence Scale—Revised (WAIS-R; Wechsler, 1981), synonyms, and analogies. Block design (WAIS-R) and card rotations assess spatial abilities. Memory tests include digit span (WAIS-R) and picture memory. Symbol digit and figure identification measure processing speed. Reliabilities for these tests range from 0.82 to 0.96 (Pedersen et al., 1992). Principal components analysis was used to construct latent factors from the individual tests within each domain: verbal, spatial, memory, and speed. Factor loadings ranged from 0.79 to 0.92 (Finkel et al., 2005). Previous comparisons of factor structure between cohorts and across testing occasions indicate that the factor structure does not vary systematically across age or time (Finkel et al., 2005). To avoid measurement variance, an invariant definition of factors at each testing occasion was created by standardizing the cognitive measures relative to the respective means and variances at IPT1. Then, loadings from the factor analyses conducted at IPT1 were used to construct the verbal, spatial, memory, and speed factors. A general cognitive ability score was created based on performance on all cognitive subtests (Finkel & Pedersen, 2004). For ease of interpretation, all factor scores were transformed to *T*-scores, using factor means and variances from IPT1.

#### Independent variables: indicators of job strain

In the 1984 SATSA-mailed questionnaire, the respondents were asked about their main lifetime occupation, “What kind of occupation did you have during the major part of your working life?” The answers were coded by Statistics Sweden according to categories from the 1980 Swedish Population and Housing Census, the same coding scheme as used in the previously validated psychosocial job exposure matrix (Johnson et al., 1990). The matrix contains separate scores for women and men for job control and job demands—measures derived from the job strain model (Karasek, 1979). *Job demands* was designed to measure psychological stress, with task pressures thought to be the best indicator of work-related stress. *Job control* is a measure of the extent to which one can use personal judgment and assert control in the workplace and is highly correlated with general decision-making authority (Karasek, 1979). Weighted averages were used to generate the scores, with a possible range of 0–10. In our study, job control scores ranged from 2.56 to 8.36 with an average of 5.09 (*SD* = 1.24; for men: range = 2.81–8.18, *M* = 5.37 ± 1.42; for women: range = 2.56–8.36, *M* = 4.82 ± 0.98), and job demands from 1.25 to 9.29 with an average of 4.73 (*SD* = 1.48; for men: range = 1.34–8.75, *M* = 4.90 ± 1.49; for women: range = 1.25–9.29, *M* = 4.57 ± 1.45). The quotient of demand over control was used to measure *job strain*, which ranged from 0.23 to 1.71 with an average of 0.95 (*SD* = 0.29; for men: range = 0.27–1.58, *M* = 0.93 ± 0.24; for women: range = 0.23–1.71, *M* = 0.97 ± 0.33).

#### Retirement age

Questions about retirement were included in SATSA questionnaires in 1987, 1990, 1993, and 2004. In addition, the same set of questions was included as part of questionnaires administered at IPT2 (1989–1991) and IPT3 (1992–1994). Included in the set of questions were items that asked respondents whether they were retired and if so, the year in which they retired. Combining this information with the birth year, we were able to calculate the retirement age for 222 individuals out of 307. Swedish retirement policy includes full-retirement benefits at age 67 without any earnings test. Between the years 1975 and 2000, the age for full benefits was 65 and was raised to 68 during 2020. The median retirement age in this sample was 64 (men = 65, women = 63), the most common retirement age was 65 (42.34% of the 222 individuals with data on retirement; 46.23% in men and 38.79% in women), and 209 (94.14%; 88.68% of men [*n* = 94] and 99.14% of women [*n* = 115]) of the participants had retired by the age of 65. Mean retirement age for the 307 participants was 63.39 (*SD* = 2.99; for men: *M* = 63.81 ± 2.80; for women: *M* = 62.99 ± 3.10) with a range of 48–75 (for men: range = 52–75; for women: range = 48–69).

#### Covariates

All covariates are known to be related to job strain and cognition. *Education* was measured in the 1984 questionnaire on a 4-point scale: 1 (*elementary school*), 2 (*vocational school*), 3 (*high school*), and 4 (*university or higher*). *Depressive symptoms* were measured at baseline assessment for each participant with the mental health subscale from the Older Americans Resources and Services Depression scale (Blazer & Williams, 1980) which includes five yes/no items. *CVD* (yes/no) was measured at baseline based on self-reports regarding the presence or absence of angina pectoris, heart attack, claudication, high blood pressure, stroke, diabetes, or any other cardiovascular dysfunction (e.g., thrombosis, tachycardia, circulation problems, heart operation, heart valve problems, and phlebitis) occurring at least once during the study period.

### Statistical Method

A growth curve model was used to examine the impact of work-related stress on cognitive aging. The structural model can be considered as a multilevel random coefficients model. The model provides an estimation of fixed effects, that is, fixed population parameters as estimated by the average growth model of the entire sample, and random effects, that is, interindividual variability in intra-individual change in growth model parameters. Growth curve models consider missing data by giving more weight to individuals with the most time points. We used a two-slope growth curve model: centering age was set at each individual’s retirement age with one linear slope before retirement age and a separate linear slope after retirement age. As a result, retirement age serves as the pivot point between the two estimated slopes. For ease of interpreting the results, job strain indicators were converted into *z*-scores, all covariates were mean centered, cognitive outcomes were expressed as *T*-scores (*M* = 50 ± 10), and time was measured in decades. The random and fixed effects parameter estimates were obtained using PROC Mixed in SAS 9.4.

Both linear and quadratic change in cognitive performance before and after retirement was examined. We compared model fit by using the −2 log-likelihood fit statistic. The results were the same when using the Akaike’s information criteria (AIC) or Bayesian information criteria (BIC) fit statistics. Adding quadratic terms for time to the models did not significantly improve model fit (*p*s > .05) and the quadratic effect of time was not statistically significant (*p*s > .05) for any of the models. Using the model estimating the association between job strain and general cognitive ability as an example, the difference in model fit corresponded to χ ^2^(16, *n* = 307) = 20.7, *p* = .190. The quadratic slope was not significant before (*p* = .839) or after (*p* = .186) retirement. Therefore, the final models estimated linear change in cognitive outcomes before/after retirement.

Of the 307 participants, 27 had missing data on depressive symptoms and four on CVD. In order to prevent further data loss, we imputed missing values for these two variables using multiple imputation procedure PROC MI in SAS with age, age at retirement, sex, and education as the auxiliary variables. The imputed values used in the main analyses reflect pooled data across 10 imputations.

We also accounted for twinness by adding a unique twin pair ID as a nesting identifier in addition to the conventional unique individual ID to all models. Although the pattern of results was similar with and without this additional adjustment, it was retained in the final models.

A two-tailed .05 *p* value was used as the threshold of statistical significance. However, given that we estimated models for five cognitive outcomes, we also applied Bonferroni correction to correct for multiple comparisons, reducing the critical *p* value to .01.

## Results

### Descriptive Statistics

Participants were followed up for an average of 5.7 waves (*SD* = 2.61), an average of 15.4 years (*SD* = 8.5 years), and up to 27 years. Descriptive characteristics of the sample and covariates are presented in Table 2. Participants were, on average, 58 years old at baseline, and 52% were women. Job strain did not correlate with age, education, depressive symptoms, or CVD; however, job control and job demands separately were correlated positively with education level.

**Table 2.** Baseline Characteristics of the Analytic Sample and Variable Intercorrelations

Variable	*M* or %	*SD*	1	2	3	4	5	6	7	8
1. Age at baseline	58.19	6.12	—							
2. Men	48%	—	0.11	—						
3. Education^a^	1.87	0.99	−0.03	0.11	—					
4. Cardiovascular disease	27%	—	0.09	0.13*	−0.08	—				
5. Depressive symptoms^b^	3.87	1.21	0.08	0.11	−0.03	−0.18**	—			
6. Job control^c^	5.09	1.24	−0.03	0.22***	0.35***	−0.08	0.09	—		
7. Job demands^c^	4.73	1.48	−0.003	0.11	0.36***	−0.06	0.13*	0.49***	—	
8. Job strain^d^	0.95	0.29	0.03	−0.06	0.09	−0.01	0.08	−0.34***	0.63***	—

^a^Education was rated on a 4-point scale from 1 (*elementary school*) to 4 (*university or higher*).

^b^Depressive symptoms were rated on a 6-point scale from 0 to 5.

^c^Scored on a scale from 0 to 10.

^d^Job strain is a ratio of demands/control.

*p < .05, **p < .01, ***p < .001.

### Job Strain and General Cognitive Ability Before, At, and After Retirement

Results from fully adjusted models that include job strain (i.e., higher demands combined with lower control) as the main predictor of general cognitive ability before, at, and after retirement are given in Table 3. First, greater job strain was associated with poorer general cognitive ability at retirement (estimate = −1.33, *SE* = 0.42, *p* = .002). For comparison, the estimate for baseline age was −0.40 (not given in Table 3). Therefore, compared to a participant with similar characteristics, retiring from a job with a job strain score higher by 1 *SD* was related to scoring on general cognitive ability as if one was about 3 years older. This association was similar in both women and men. Greater job strain was also associated with less improvement of general cognitive ability before retirement (estimate = −0.79, *SE* = 0.29, *p* = .007), as well as slower decline after retirement (estimate = 0.37, *SE* = 0.16, *p* = .020), although this last result did not reach statistical significance after Bonferroni correction for multiple comparisons. For comparison, in fully adjusted models, age alone was associated with improvement by 0.11 points per decade before retirement and a decline of 0.06 points per decade after retirement.

**Table 3.** Relationship Between Job Strain and General Cognitive Ability Before, At, and After Retirement

General cognitive ability	Total, *N* = 307			Women, *N* = 159			Men, *N* = 148		
	Est.	*SE*	*p*	Est.	*SE*	*p*	Est.	*SE*	*p*
Intercept^a^	**56.97**	0.47	—	57.19	0.65	—	56.76	0.74	—
Change before retirement	1.17	0.62	.059	**1.98**	0.90	.027	0.70	0.85	.411
Change after retirement	**−3.18**	0.17	<.001	**−3.19**	0.25	<.001	**−3.14**	0.26	<.001
Job strain^b^	**−1.33**	0.42	.002	**−1.22**	0.52	.020	**−1.86**	0.73	.011
Job strain × Preretirement change	**−0.79**	0.29	.007	**−1.09**	0.39	.005	0.02	0.47	.974
Job strain × Postretirement change	**0.37**	0.16	.020	0.36	0.23	.120	**0.66**	0.25	.008

*Notes:* Age in years (per decade) was the time scale, age of retirement was the pivot point between the two estimated slopes, Est. = unstandardized regression coefficient, *SE* = standard error of measurement, *p* < .05 in bold. Adjusted for age, sex, education, depressive symptoms, cardiovascular factors, and twinness. Because data were nested by both individual and twin pair, the *p* values for the intercept are not calculated.

^a^Cognitive score, expressed in *T*-score units, at age of retirement.

^b^Cross-sectional association between job strain and cognition at the intercept.

In the sex-stratified analyses, we observed that the association between greater job strain and smaller gains in general cognitive ability before retirement was only observed in women (estimate = −1.09, *SE* = 0.39, *p* = .005; Table 3). The slower decline after retirement in general cognitive ability as a function of greater job strain was only significant in men (estimate = 0.66, *SE* = 0.25, *p* = .008).

**Figure 1.**
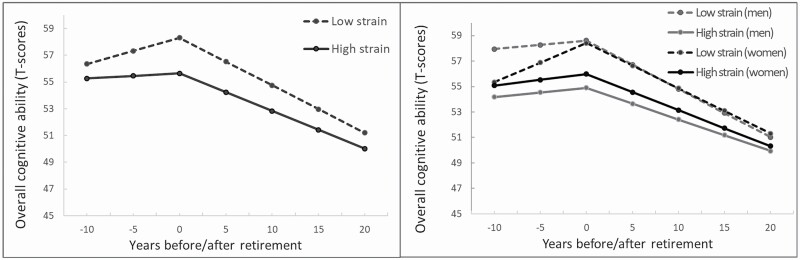
(**A** and **B**) Graphical illustration of the association between job strain and trajectory of change in general cognitive ability before and after retirement adjusting for age, sex, education, depressive symptoms, cardiovascular factors, and twinness. The solid line represents job strain score 1 *SD* above the mean; the dashed line represents job strain 1 *SD* below the mean. (A) The figure illustrates the total analytic sample and (B) women and men separately.

The results presented above are illustrated in Figure 1A for the entire sample and in Figure 1B for men/women separately.

### Job Strain and Change in Specific Cognitive Domains Before, At, and After Retirement

Results for the fully adjusted associations between job strain indicators and cognitive aging before, at, and after retirement are given in Table 4. Greater job strain was associated with poorer memory scores (estimate = −1.22, *SE* = 0.45, *p* = .007), speed (estimate = −1.11, *SE* = 0.44, *p* = .012), and spatial ability (estimate = −0.96, *SE* = 0.47, *p* = .043) at retirement. The last two results did not reach statistical significance after the Bonferroni correction. A similar pattern was observed between greater job strain at retirement and poorer scores on verbal ability, although this result did not reach statistical significance (estimate = −0.66, *SE* = 0.42, *p* = .120). The same patterns were also observed in the sex-stratified analyses; however, the association between greater job strain and poorer speed at retirement was stronger for men than women.

**Table 4.** Relationship Between Job Strain and Cognitive Ability Before, At, and After Retirement

	Memory			Speed			Verbal ability			Spatial ability^c^		
Total	Est.	*SE*	*p*	Est.	*SE*	*p*	Est.	*SE*	*p*	Est.	*SE*	*p*
Intercept^a^	54.58	0.51	—	56.41	0.52	—	55.28	0.45	—	55.66	0.54	—
Change before retirement	1.06	1.03	.302	0.04	0.93	.967	2.04	0.54	<.001	−0.25	0.91	.788
Change after retirement	−1.57	0.28	<.001	−4.68	0.25	<.001	−0.65	0.14	<.001	−2.89	0.26	<.001
Job strain^b^	−1.22	0.45	.007	−1.11	0.44	.012	−0.66	0.42	.120	−0.96	0.47	.043
Job strain × Preretirement change	−0.69	0.47	.144	−0.40	0.43	.358	−0.32	0.25	.192	−0.68	0.42	.109
Job strain × Postretirement change	0.67	0.25	.007	0.23	0.22	.299	0.12	0.12	.321	0.29	0.23	.204
*Women*												
Intercept^a^	55.62	0.70	—	57.93	0.74	—	54.68	0.64	—	54.18	0.75	—
Change before retirement	0.73	1.38	.595	1.64	1.35	.223	2.04	0.78	.009	1.00	1.25	.427
Change after retirement	−1.81	0.36	<.001	−4.94	0.35	<.001	−0.49	0.20	.014	−2.81	0.35	<.001
Job strain^b^	−1.22	0.56	.029	−0.89	0.56	.112	−0.57	0.54	.289	−0.97	0.59	.098
Job strain × Preretirement change	−0.76	0.56	.180	−0.24	0.56	.664	−0.64	0.32	.047	−1.35	0.52	.010
Job strain × Postretirement change	0.85	0.32	.008	−0.01	0.30	.975	0.30	0.17	.087	0.45	0.32	.136
*Men*												
Intercept^a^	53.64	0.79	—	54.86	0.77	—	55.97	0.66	—	57.02	0.82	—
Change before retirement	1.72	1.55	.269	−0.70	1.26	.578	2.21	0.75	.003	−1.27	1.36	.352
Change after retirement	−1.19	0.47	.012	−4.39	0.37	<.001	−0.86	0.22	<.001	−3.10	0.42	<.001
Job strain^b^	−1.66	0.80	.037	−1.67	0.74	.024	−1.28	0.68	.061	−1.09	0.81	.180
Job strain × Preretirement change	−0.54	0.86	.527	−0.45	0.70	.522	0.34	0.42	.407	0.61	0.75	.416
Job strain × Postretirement change	1.12	0.45	.013	0.70	0.36	.050	0.14	0.21	.500	0.45	0.40	.254

*Notes:* Age in years (per decade) was the time scale, age of retirement was the pivot point, Est. = unstandardized regression coefficient, *SE* = standard error of measurement, *p* < .05 in bold. Adjusted for age, sex, education, depressive symptoms, cardiovascular factors, and twinness. Because data were nested by both individual and twin pair, the *p* values for the intercept are not calculated.

^a^Cognitive *T*-score at the age of retirement.

^b^Cross-sectional association between job strain and cognition at the intercept.

^c^Analyses for spatial ability combining men and women could not be adjusted for depressive symptoms and cardiovascular factors due to lack of model convergence.

No statistically significant association between job strain and change in memory, speed, verbal ability, or spatial ability before retirement was found for the entire sample. However, sex-stratified analyses indicated that greater job strain was significantly associated with less improvement in spatial ability (estimate = −1.35, *SE* = 0.52, *p* = .010) and verbal ability (estimate = −0.64, *SE* = 0.32, *p* = .047) preretirement among women but not among men. Note that both results are at or above the Bonferroni corrected *p* value of .01. Supplementary analyses indicated that the association between job strain and preretirement verbal and spatial ability was mainly driven by job control scores (Supplementary Table 1).

In analyses with the entire sample, greater job strain was associated with a slower decline in memory after retirement (estimate = 0.67, *SE* = 0.25, *p* = .007), a pattern that was also found among women (estimate = 0.85, *SE* = 0.32, *p* = .008) and men (estimate = 1.12, *SE* = 0.45, *p* = .013), although the result for men was not significant after Bonferroni correction. Supplementary analyses indicated that the association between job strain and postretirement memory was foremost driven by job control (Supplementary Tables 1 and 2).

## Discussion

This longitudinal study investigated the relationship between job strain and cognitive change in four domains (verbal, spatial, memory, and speed) and general cognitive ability with up to 27 years of follow-up. The unique features of the SATSA data include data collection well before age 65 and a long follow-up using IPT, which allowed us to use a two-slope growth model centered around the age at retirement that provides a simultaneous assessment of change in cognitive ability as a function of job strain pre- and postretirement. Overall, we found that, after adjusting for study covariates, greater job strain was associated with less improvement in general cognitive ability prior to retirement, worse memory, speed, spatial ability, and general cognitive ability at retirement age, and a slower decline in memory and general cognitive ability in the years following retirement (more so for men). The smaller gains in general cognitive ability prior to retirement were more pronounced for spatial and verbal ability in women.

These findings highlight the importance of work-related stress in cognitive aging. Specifically, individuals retiring from jobs with high levels of stress, those having jobs with less control over work tasks and decision-making authority in combination with greater task pressure (i.e., greater job strain; Karasek, 1979), entered retirement with a significant cognitive disadvantage in all measured domains and general cognitive ability. For example, comparing the estimates for job strain in relation to general cognitive ability with age, a person with job strain higher by 1 *SD* performed at an overall cognitive level indicative of someone with the same personal characteristics (but lower job strain) who was about 3 years older (job strain estimate = −1.33 vs. age-at-retirement estimate = −0.40). In the same fully adjusted models, the longitudinal results indicated that being 1 year older was a related improvement in the general cognitive score by 0.11 points/decade preretirement and a decline by 0.06 points/decade postretirement. In comparison, job strain higher by 1 *SD* was related to cognitive scores improving by 0.79 points/decade *less* preretirement but also worsening at a *slower* rate by 0.37 points/decade postretirement. These results underscore the role of job strain in cognitive aging.

The results also build on previous work suggesting that job strain may accelerate cognitive aging (Andel et al., 2015; Nexø et al., 2016; Sabbath et al., 2016; Then et al., 2014). Stress derived from work is one of the major stressors in adult life and plays a prominent role in shaping health in working ages (Mezuk et al., 2011; Nyberg et al., 2013) as well as in postretirement life (Andel et al., 2015; Pan et al., 2019; Sindi et al., 2016). Stress at work may not only reduce the likelihood of a prolonged working life (Elovainio et al., 2005), which is often proposed as part of the solution to meeting the economic demands of an aging population, it may also cause negative long-term effects on the body via biochemical pathways that reach far into postretirement life. When experiencing high stress levels over a long period of time, the hypothalamus–pituitary–adrenal (HPA) axis activity is increased, resulting in a greater release of the stress hormone cortisol (Wolf, 2009). Elevated levels of cortisol reduce hippocampal activity, which may adversely affect cognitive functioning (Lupien et al., 2007).

Although job demands are considered stressful (Karasek, 1979), the rather weak findings related to demands in this study may reflect the double nature of demands. That is, demands not only represent stress but also intellectual stimulation and engagement at work, which may have favorable effects on cognitive function (Nexø et al., 2016; Then et al., 2014).

The pattern of results was generally similar for women and men, which is in line with earlier research (Nexø et al., 2016; Then et al., 2014). However, preretirement, greater job strain was related to smaller gains in general cognitive ability in women, whereas there was no difference in overall cognitive change as a function of job strain for men. The result for women appeared to be driven by the stronger associations between greater job strain and less improvement in spatial and verbal ability preretirement. The double burden of job strain and household/family duties often experienced in women (Krantz et al., 2005) may partially explain why women are more negatively affected by work-related stress during working years.

Although there is no consensus from earlier research about whether extended working lives are beneficial or not for cognitive function (Bonsang et al., 2012; Coe et al., 2012), this is likely due to the heterogeneity in different occupations and working conditions. We found that greater job strain modifies trajectories of cognitive aging before/after retirement. In the overall sample, there was an improvement in cognitive performance before retirement and decline after retirement. With greater job strain, there may be lower cognitive function regardless of age, but also smaller cognitive gains preretirement and slightly slower cognitive decline postretirement.

Analyses with memory as the outcome mimicked this pattern of results more so than speed, spatial ability, or verbal ability. A potential explanation for our findings is that when people retire from a more stressful work environment, the negative effect that work-related stress on cognition may dissipate (Fisher et al., 2017), reducing stress-induced effects on the hippocampus, the center for memory and learning, by less activation of the HPA axis (Kim et al., 2015). However, this finding was in contrast to earlier research where greater job strain was associated with an accelerated decline in episodic memory after retirement (Andel et al., 2015). Of note is that Andel et al. used data from the U.S.-based Health and Retirement Study. It may be that the phone-based only cognitive assessment could not fully capture more subtle preretirement differences in cognitive aging. It may also be that cross-national differences contributed to the divergent results for this Sweden-based study and the study based in the United States where an average retiree may experience more postretirement stress through relatively low financial security. More research is needed in this area.

The study findings should be interpreted with caution. SATSA is based on a representative sample of the population in Sweden (Finkel & Pedersen, 2004). Still, all studies including older adults are prone to selective survival. To minimize nonrandom dropouts, nurses visited the participants in their current residence providing the possibility to continue to be part of the SATSA sample even after entry into care or onset of illness. Moreover, growth curve models allow people to have missing outcome data at some of the timepoints and give more weight to observations from those participating in more waves, hence, being less sensitive to selective attrition. Studies investigating work characteristics are also vulnerable to the healthy worker effect, that is, those working are probably healthier than those not working. However, the healthy worker effect, together with selective survival, probably leads to an underestimation of the true association between work-related stress and cognitive ability. Finally, our rather strict inclusion criteria resulted in a fairly small sample size. Still, we believe such criteria are needed to maintain proper internal validity. The unique strength of this data set is that it is one of the few data sets in the world with comprehensive cognitive data collected in-person both before and after retirement, as well as information about work characteristics and age at retirement.

A strength of this study was the inclusion of multiple cognitive domains. This provided a more nuanced picture of the relationship between work-related stress and cognitive change. Furthermore, although we only measured work-related stress once during working life, the question asked specifically about main lifetime occupation. Combined with a relatively low occupational mobility in this cohort, our estimates should apply well to the entire working life. Also, nonwork stressors may interact with workplace stressors in terms of determining overall levels of chronic stress and influencing cognitive ability. On that same note, individual differences in stress exposures and responses may be missed when using an occupation-based measure of work stressors instead of measuring people’s perception of possibly stressful work situations. However, there is some evidence suggesting that people have physiological stress responses to objectively stressful work environment, regardless of the subjective perception of stress (Häusser et al., 2011). Moreover, Theorell (2000) suggests that even though there are individual differences in reactions to workplace stressors, investigating occupation-based stressors may still be valuable because individual differences, in most cases, are random and somewhat equally distributed between different workplaces. Identifying the most significant stressors in the work environment associated with cognitive change may help target interventions to prevent cognitive decline. Still, it is important to note that we cannot exclude the risk of reverse causation, whereby cognitive abilities preselect participants into certain occupations.

In conclusion, the results show the importance of work-related stress in cognitive aging. Findings highlight that more stressful occupations may have a negative influence on overall cognitive functioning prior to and at the time of retirement, but could slightly reduce age-related cognitive decline postretirement. These results provide evidence that stressors in the work environment should be seen as important targets for intervention.

## Funding

This work was supported by the Marcus and Marianne Wallenberg Foundation (grant number MMW 2016.0081) and Swedish Council for Working Life and Social Research (Forte; grant number 2019-01141 to C. Nilsen). SATSA is supported by the National Institute of Aging (grant numbers AG04563, AG10175, and AG08724); the MacArthur Foundation Research Network on Successful Aging; Swedish Research Council (grant numbers 825-2007-7460, 825-2009-6141, and 825-3011-6182); and the Swedish Council for Working Life and Social Research (Forte; grant numbers 97:0147:1B, 2009-0795).

## Conflict of Interest

None declared.

## Data Availability

Data, analytic methods, and materials will be made available to other researchers if requested. The study has not been preregistered.

## Supplementary Material

gbab033_suppl_Supplementary_Table_1

gbab033_suppl_Supplementary_Table_2
