# Supplementary material for: Job Strain and Trajectories of Cognitive Change Before and After Retirement
Source: J Gerontol B Psychol Sci Soc Sci. 2021 Feb 24;76(7):1313–22. doi: 10.1093/geronb/gbab033 (PMC8363035; doi:10.1093/geronb/gbab033)
Supplement: gbab033_suppl_Supplementary_Table_1 [file gbab033_suppl_supplementary_table_1.pdf]

Supplementary Table 1 Relationship between job control and cognition before, at, and after retirement

	Total sample			Women			Men		
**Cognitive domain**	Est.	SE	p-value	Est.	SE	p-value	Est.	SE	p-value
**General cognitive ability**									
Intercept[a]	56.96	0.48	-	57.38	0.66	-	56.50	0.75	-
Change before retirement	**1.30**	0.62	.035	**2.38**	0.90	.009	1.07	0.84	.200
Change after retirement	**-3.20**	0.17	<.001	**-3.36**	0.25	<.001	**-3.01**	0.26	<.001
Job control[b]	**1.66**	0.44	<.001	**1.87**	0.77	.015	**1.82**	0.52	<.001
Job control x pre-retirement change	-0.36	0.30	.231	**2.03**	0.67	.003	**-1.03**	0.31	<.001
Job control x post-retirement change	**-0.43**	0.17	.014	**-0.81**	0.30	.007	-0.39	0.22	.073
**Memory**									
Intercept[a]	54.59	0.50	-	55.72	0.71	-	53.28	0.78	-
Change before retirement	1.09	1.02	.287	1.08	1.39	.436	2.03	1.54	.187
Change after retirement	**-1.58**	0.28	<.001	**-2.02**	0.37	<.001	-0.84	0.47	.075
Job control[b]	**1.66**	0.48	<.001	0.93	0.83	.260	**2.19**	0.58	<.001
Job control x pre-retirement change	-0.04	0.49	.930	1.82	1.01	.072	-0.53	0.56	.343
Job control x post-retirement change	**-0.82**	0.27	.003	-0.78	0.42	.062	**-1.34**	0.39	<.001
**Speed**									
Intercept[a]	56.41	0.52	-	58.18	0.75	-	54.70	0.78	-
Change before retirement	0.17	0.92	.850	1.60	1.36	.240	-0.15	1.25	.903
Change after retirement	**-4.69**	0.25	<.001	**-4.98**	0.35	<.001	**-4.27**	0.37	<.001
Job control[b]	**1.34**	0.46	.004	**1.99**	0.82	.016	**1.20**	0.53	.025
Job control x pre-retirement change	**-0.92**	0.44	.038	0.47	0.99	.633	**-1.21**	0.46	.008
Job control x post-retirement change	-0.23	0.24	.342	-0.29	0.40	.469	-0.27	0.31	.373
**Verbal ability**									
Intercept[a]	55.30	0.44	-	55.04	0.64	-	**55.75**	0.66	-
Change before retirement	**2.08**	0.54	<.001	**2.30**	0.78	.003	**2.33**	0.75	.002
Change after retirement	**-0.65**	0.14	<.001	**-0.63**	0.20	.002	**-0.84**	0.22	<.001
Job control[b]	**1.61**	0.44	<.001	**2.11**	0.78	.007	**1.54**	0.50	.002
Job control x pre-retirement change	-0.03	0.26	.923	**1.35**	0.57	.018	-0.50	0.27	.066

Job control x post-retirement change	-0.17	0.14	.214	**-0.64**	0.23	.005	-0.01	0.18	.949
**Spatial ability**									
Intercept[a]	55.66	0.54	-	**54.29**	0.77	-	56.96	0.83	-
Change before retirement	0.01	0.91	.999	1.55	1.26	.221	-1.08	1.36	.426
Change after retirement	**-2.95**	0.26	<.001	**-2.97**	0.35	<.001	**-3.11**	0.42	<.001
Job control[b]	0.63	0.50	.205	1.23	0.87	.157	0.41	0.61	.502
Job control x pre-retirement change	-0.31	0.44	.478	**2.43**	0.92	.008	**-1.14**	0.50	.022
Job control x post-retirement change	0.00	0.26	.998	-0.75	0.42	.075	0.30	0.35	.403

Note. Age in years (per decade) was the time scale, age of retirement was the pivot point between the two estimated slopes, Est.= Unstandardized regression coefficient, SE = standard error of measurement, $p < .05$ in bold. Adjusted for age, sex, education, depressive symptoms, cardiovascular factors, and twiness. Because data were nested by both individual and twin pair, the p-values for the intercept are not calculated. [a]Cognitive T-score at age of retirement. [b]Cross-sectional association between job control and cognition at the intercept.
