# Supplementary material for: Job Strain and Trajectories of Cognitive Change Before and After Retirement
Source: J Gerontol B Psychol Sci Soc Sci. 2021 Feb 24;76(7):1313–22. doi: 10.1093/geronb/gbab033 (PMC8363035; doi:10.1093/geronb/gbab033)
Supplement: gbab033_suppl_Supplementary_Table_2 [file gbab033_suppl_supplementary_table_2.pdf]

Supplementary Table 2 Relationship between job demands and cognition before, at, and after retirement

	Total sample			Women			Men		
**Cognitive domain**	Est.	SE	p-value	Est.	SE	p-value	Est.	SE	p-value
**General cognitive ability**									
Intercept[a]	56.95	0.48	-	57.03	0.66	-	56.74	0.75	-
Change before retirement	1.20	0.62	.051	**2.00**	0.91	.028	0.57	0.84	.495
Change after retirement	**-3.21**	0.17	<.001	**-3.23**	0.26	<.001	**-3.07**	0.26	<.001
Job demands[b]	-0.02	0.45	.960	-0.77	0.66	.244	0.62	0.64	.331
Job demands x pre-retirement change	**-0.93**	0.29	.002	-0.59	0.51	.246	**-1.10**	0.34	.001
Job demands x post-retirement change	0.06	0.20	.744	0.13	0.28	.651	0.03	0.28	.903
**Memory**									
Intercept[a]	54.58	0.51	-	55.45	0.69	-	53.54	0.80	-
Change before retirement	1.02	1.03	.319	0.85	1.39	.539	1.50	1.54	.329
Change after retirement	**-1.61**	0.28	<.001	**-1.75**	0.37	<.001	**-0.99**	0.48	.040
Job demands[b]	-0.04	0.49	.930	-1.17	0.69	.093	0.90	0.69	.192
Job demands x pre-retirement change	-0.83	0.47	.080	-0.24	0.74	.747	-1.18	0.63	.061
Job demands x post-retirement change	0.19	0.29	.513	0.72	0.37	.055	-0.48	0.50	.338
**Speed**									
Intercept[a]	56.39	0.53	-	57.79	0.75	-	54.88	0.79	-
Change before retirement	-0.02	0.92	.980	1.58	1.36	.243	-0.66	1.24	.597
Change after retirement	**-4.67**	0.25	<.001	**-4.95**	0.35	<.001	**-4.34**	0.38	<.001
Job demands[b]	-0.05	0.47	.912	-0.25	0.68	.714	0.23	0.64	.725
Job demands x pre-retirement change	-1**.02**	0.43	.018	-0.20	0.73	.781	**-1.54**	0.51	.003
Job demands x post-retirement change	0.03	0.26	.895	-0.09	0.35	.807	0.18	0.40	.655
**Verbal ability[c]**									
Intercept[a]	55.27	0.45	-	54.63	0.64	-	55.97	0.66	-
Change before retirement	**2.12**	0.54	<.001	**2.06**	0.78	.009	**2.11**	0.74	.005
Change after retirement	**-0.68**	0.14	<.001	**-0.50**	0.20	.013	**-0.86**	0.22	<.001
Job demands[b]	0.55	0.45	.223	0.33	0.69	.634	0.64	0.60	.283
Job demands x pre-retirement change	-0.37	0.25	.138	-0.20	0.42	.625	-0.27	0.30	.370

Job demands x post-retirement change	0.13	0.15	.376	0.04	0.20	.827	0.09	0.23	.693
**Spatial ability**									
Intercept[a]	55.64	0.55	-	54.05	0.75	-	57.07	0.82	-
Change before retirement	-0.07	0.91	.937	1.03	1.27	.417	-1.44	1.34	.286
Change after retirement	**-2.93**	0.26	<.001	**-2.82**	0.36	<.001	**-3.15**	0.42	<.001
Job demands[b]	-0.48	0.50	.336	-0.90	0.73	.214	-0.16	0.71	.818
Job demands x pre-retirement change	-0.76	0.42	.073	-0.83	0.68	.224	-0.85	0.55	.123
Job demands x post-retirement change	0.32	0.29	.269	0.33	0.39	.394	0.56	0.45	.218

Note. Age in years (per decade) was the time scale, age of retirement was the pivot point between the two estimated slopes, Est.= Unstandardized regression coefficient, SE = standard error of measurement, $p < .05$ in bold. Adjusted for age, sex, education, depressive symptoms, cardiovascular factors, and twiness. Because data were nested by both individual and twin pair, the p-values for the intercept are not calculated. [a]Cognitive T-score at age of retirement. [b]Cross-sectional association between job demands and cognition at the intercept. [c]Analyses for verbal ability combining men and women could not be adjusted for depressive symptoms and cardiovascular factors due to lack of model convergence.
